# Eosinophilia and IgE Elevation: An Uncommon Toxocara Infection

**DOI:** 10.7759/cureus.77965

**Published:** 2025-01-25

**Authors:** Jorge Reis, Francisca Carmo, João Miranda, Andreia P Seixas, Margarida Mota

**Affiliations:** 1 Internal Medicine, Unidade Local de Saúde Gaia/Espinho, Vila Nova de Gaia, PRT

**Keywords:** albendazole, case report, eosinophilia, ige, parasitic infection, toxocara, toxocariasis

## Abstract

Eosinophilia and elevated immunoglobulin E (IgE) levels are common indicators of parasitic infections, allergic reactions, or autoimmune conditions.

In this clinical report, we present the case of a 56-year-old woman who was observed in a follow-up consultation, whose laboratory investigations revealed significant eosinophilia (5.52 × 10E^3^/uL) and elevated serum IgE levels (19253.00 IKU/L). The patient had no symptoms and the physical examination was unremarkable. An abdominal ultrasound was performed showing no abnormalities. Further diagnostic workup, including serological tests and imaging, led to the identification of Toxocariasis, a parasitic infection caused by the roundworm larvae of Toxocara canis, a parasitic nematode commonly transmitted via contaminated soil with animal feces. Upon further investigation, the patient was found to be diagnosed with Pica disorder which is characterized by the persistent craving and consumption of non-food substances, such as dirt, clay, chalk, paper, hair, or paint. After initiating antiparasitic treatment with albendazole, the patient showed a gradual normalization of eosinophil counts and IgE levels.

This case highlights the importance of considering toxocariasis infection in patients presenting with eosinophilia and elevated IgE, even in an urban setting and areas of low prevalence of Toxocara such as Europe. In this case, the patient had no symptoms and four months prior laboratory work was without abnormalities. Early diagnosis and treatment are crucial for favorable outcomes.

## Introduction

Eosinophilia and elevated immunoglobulin E (IgE) levels are laboratory markers commonly associated with different clinical conditions, including allergic disorders, parasitic infections, and certain hematological malignancies. IgE level is an immunological marker that reflects the activation of the immune system in response to a specific stimulus, often associated with type 2 hypersensitivity reactions, where IgE antibodies mediate the immune response to allergens [1].

When eosinophilia and elevated IgE are both present, it is crucial to consider conditions involving dysregulated immune responses, such as allergic reactions, hyper-IgE syndrome, eosinophilic granulomatosis with polyangiitis (Churg-Strauss syndrome), or other systemic inflammatory disorders. Furthermore, eosinophilia and elevated IgE levels may also be seen in less frequent and more complex conditions, such as autoimmune diseases, malignancies (e.g., eosinophilic leukemia or lymphoma), or vasculitis. Although these findings are typically linked to well-established pathophysiological mechanisms, their occurrence in isolated or unusual clinical presentations warrants detailed investigation [2].

Toxocara is a genus of parasitic roundworms (nematodes) that infect both animals and humans, with infections occurring globally but more commonly in developing countries. The most common species affecting humans are Toxocara canis and Toxocara cati. Humans are accidental hosts, typically infected through ingestion of parasite eggs found in soil, contaminated food, or water. The most common clinical features include abdominal pain, hepatomegaly, anorexia, nausea, vomiting, lethargy, sleep and behavioral disturbances, pneumonia, cough, wheezing, pharyngitis, cervical lymphadenitis, headache, limb pain, and fever [3]. Enzyme-linked immunosorbent assay (ELISA) for Toxocara-specific antibodies is the main diagnostic tool, although tissue biopsy provides the definitive diagnosis; however, due to its invasive nature, it is often dispensable [4, 5].

Eosinophilia and elevated immunoglobulin E (IgE) levels can signal a variety of conditions, but the underlying cause may not be immediately apparent, requiring a thorough workup to rule out rare or unconventional causes. This report highlights a case where these markers, absence of symptoms, and appropriate context led to the diagnosis of toxocariasis, a parasitic infection often overlooked in clinical practice.

## Case presentation

We present a case of a 56-year-old female patient originally from Angola and residing in Portugal since 1975 with a history of successfully treated hepatitis C with glecaprevir/pibrentasvir four years earlier, past history of intravenous drug use (heroin), arterial hypertension, and a treated syphilis. She was on a regimen of methadone, omeprazole, escitalopram, lorazepam, olanzapine, and mirtazapine.

Significant abnormalities were noted during a routine laboratory workup in a regular infectious disease consultation, including elevated eosinophil count and IgE levels in repeated measures. She had no physical complaints and the physical examination was unremarkable, with no evidence of hepatosplenomegaly or lymphadenopathy. The patient denied potential causes of eosinophilia and elevated IgE levels, including common allergies (e.g., allergic rhinitis, asthma, atopic dermatitis), symptoms compatible with parasitic infections, or constitutional symptoms that could be suspicious of hematological conditions (e.g., eosinophilic leukemia, lymphoma).

**Figure 1:**
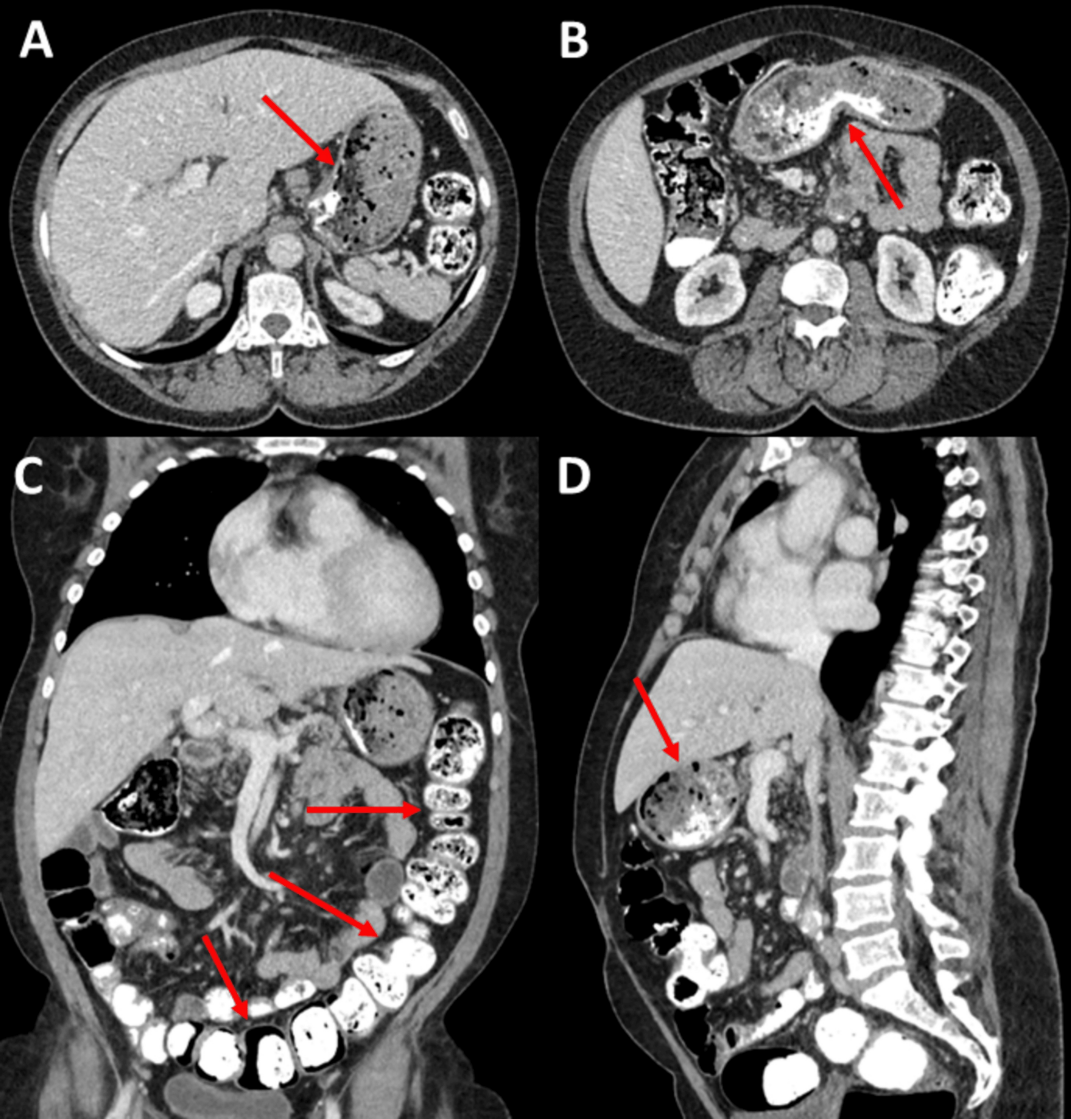
Abdominal and Thoracic CT Scan Axial (A, B), coronal (C), and sagittal (D) CT images demonstrate high-density material in the gastrointestinal tract (arrows), consistent with sand ingestion related to pica behavior. The images highlight the presence of radiopaque content within the stomach and intestines, which correlates with the patient's history of ingesting sand during periods of stress or anxiety.

Despite the patient's asymptomatic presentation, these laboratory results prompted further investigation to determine the underlying cause of the elevated eosinophil count and IgE levels. An abdominal ultrasound showed no abnormalities, but a CT scan revealed a pulmonary embolism and high-density material of unknown origin filling almost the entire digestive tract lumen (Figure 1). When confronted with these findings, the patient disclosed a long-standing history of Pica disorder, ingesting sand (from plant vases, cat boxes and ashtrays) during periods of heightened anxiety or stress.

Empiric treatment with albendazole 400 mg daily for five days was initiated. Anticoagulation therapy with apixaban was also started to address the pulmonary embolism. The patient was counseled about the health risks associated with Pica disorder, and the importance of managing her anxiety to prevent further sand ingestion was emphasized.

Serological testing for Toxocara returned positive for IgG (both ELISA and Immunoblot), confirming the diagnosis of toxocariasis (Table 1). Mutation testing for FIP1L1-PDGFRA was negative, effectively ruling out chronic eosinophilic leukemia. This case highlights the diagnostic challenges posed by atypical presentations of eosinophilia and the value of considering parasitic infections in patients with relevant exposures.

**Table 1:** Laboratory Results The table summarizes the patient's laboratory results, including hematological parameters, immunoglobulin levels, and serological findings for Toxocara species. Notable abnormalities include significant eosinophilia, elevated serum IgE levels, and positive Toxocara IgG.

Laboratory Parameter	Result	Reference Range
Hemoglobin (g/dL)	15.2	12.0-16.0
Leukocyte count (×10^3^/µL)	10.33	3.6-11.0
Neutrophils	2.04 (19.8%)	1.3-8.8
Eosinophils	5.52 (53.4%)	0.0-0.5
Basophils	0.03 (0.3%)	0.0-0.2
Lymphocytes	2.42 (23.4%)	1.0-4.8
Monocytes	0.31 (3.0%)	0.1-0.8
Platelet count (×10^3^/µL)	314	150-440
C-reactive protein (mg/dL)	0.37	0-0.5
IgE (IKU/L)	19,253.00	0-114
IgG (mg/dL)	2760	680-1450
IgA (mg/dL)	316	83-407
IgM (mg/dL)	137	34-214
Toxocara sp IgG (U)	8.9	Negative <0.9, Inconclusive 0.9-1.1, Positive >1.1

## Discussion

Eosinophilia and elevated IgE levels are well-established immunological markers commonly associated with a broad range of diseases, from common allergic conditions to rare autoimmune disorders and hematologic malignancies such as chronic eosinophilic leukemia [1].

Parasitic infections should be considered in patients presenting with eosinophilia and elevated IgE, particularly in cases with a history of behavior such as Pica disorder. A study by Belmesk et al. (2022) underscores the importance of early recognition of toxocariasis, particularly in patients with a history of ingesting soil or sand, as was the case with our patient. Toxocariasis infections are often associated with eosinophilia and elevated IgE, with clinical manifestations ranging from gastrointestinal symptoms to respiratory and neurological involvement [2]. Pica disorder is a significant risk factor for parasitic infections, especially those caused by Toxocara, due to the ingestion of parasite eggs from contaminated soil [3].

Serological tests like ELISA are a sensitive and specific diagnostic tool for detecting toxocariasis. This non-invasive diagnostic approach is particularly beneficial in cases with unusual clinical presentations, where other diagnostic options may be less accessible or invasive [4]. Our case also benefited from this diagnostic approach, confirming the diagnosis of toxocariasis through positive IgG serology. The diagnosis of chronic eosinophilic leukemia was ruled out due to the negative FIP1L1-PDGFRA mutation test results.

Furthermore, imaging studies, particularly CT scans, play an essential role in identifying potential complications or associated conditions. The presence of a concomitant pulmonary embolism added complexity to the diagnosis but was managed appropriately with anticoagulation therapy [5].

Albendazole has been shown to be an effective therapeutic option for toxocariasis. A retrospective study by Magnaval et al. (2022) demonstrated the efficacy of albendazole in treating human toxocariasis, with significant clinical improvement and a lower incidence of adverse effects compared to alternative therapies [6].

The patient was referred for psychiatric consultation to address the underlying anxiety and stress contributing to her Pica disorder behavior. Following treatment with albendazole for toxocariasis, she showed favorable clinical progress. There were no signs of cytopenias or abnormalities in other white blood cell lineages, and the eosinophil count normalized.

Follow-up visits confirmed her recovery, with continued management of her psychiatric symptoms to prevent relapse of Pica and ensure long-term behavioral stability. Given the resolution of the parasitic infection and normalization of laboratory values, the prognosis was good, though ongoing psychological support is needed to address the root cause of her Pica behavior [7].

## Conclusions

This case underscores the importance of considering parasitic infections, such as toxocariasis, in patients with eosinophilia and elevated IgE levels, even in the absence of overt symptoms. The finding of solid material in the CT scan and the patient’s history of Pica disorder was a key factor in reaching the diagnosis.

This highlights the necessity of maintaining a broad differential diagnosis in atypical clinical presentations, as eosinophilia and elevated IgE can be indicative of a wide array of conditions. The successful management of toxocariasis with albendazole and the resolution of laboratory abnormalities emphasize the importance of early detection and targeted treatment. Addressing underlying behavioral issues with appropriate psychiatric support is crucial for preventing recurrence and ensuring long-term health.
